# Descending control of nociception in insects?

**DOI:** 10.1098/rspb.2022.0599

**Published:** 2022-07-13

**Authors:** Matilda Gibbons, Sajedeh Sarlak, Lars Chittka

**Affiliations:** ^1^ School of Biological and Behavioural Sciences, Queen Mary University of London, London E1 4NS, UK; ^2^ Department of Plant Protection, College of Agriculture and Natural Resources, University of Tehran, 31587-77871, Karaj, Iran

**Keywords:** adaptation, ethics, insects, modulation, nociception, pain

## Abstract

Modulation of nociception allows animals to optimize chances of survival by adapting their behaviour in different contexts. In mammals, this is executed by neurons from the brain and is referred to as the descending control of nociception. Whether insects have such control, or the neural circuits allowing it, has rarely been explored. Based on behavioural, neuroscientific and molecular evidence, we argue that insects probably have descending controls for nociception. Behavioural work shows that insects can modulate nocifensive behaviour. Such modulation is at least in part controlled by the central nervous system since the information mediating such prioritization is processed by the brain. Central nervous system control of nociception is further supported by neuroanatomical and neurobiological evidence showing that the insect brain can facilitate or suppress nocifensive behaviour, and by molecular studies revealing pathways involved in the inhibition of nocifensive behaviour both peripherally and centrally. Insects lack the endogenous opioid peptides and their receptors that contribute to mammalian descending nociception controls, so we discuss likely alternative molecular mechanisms for the insect descending nociception controls. We discuss what the existence of descending control of nociception in insects may reveal about pain perception in insects and finally consider the ethical implications of these novel findings.

## 1. Descending control of nociception

Nociception is the detection of potentially or actually damaging stimuli, which is mediated by specialized receptors: nociceptors [1]. Nociception can be accompanied by the feeling of pain, which is a negative subjective experience generated by the brain [2,3]. Nociception and/or pain can be inhibited or facilitated (modulated) by descending neurons from the brain (including the brainstem in vertebrates) called ‘the descending pain controls' [4,5]. There are distinctive mechanisms behind modulation of nociception and modulation of pain, and recent studies have uncovered that certain contexts or stimuli can modulate pain report while keeping nociceptive reflexes unchanged [6–8], and vice versa, where nociceptive reflexes are modulated but pain report is unchanged [9]. Therefore, in animals where the distinction of pain and nociception has not yet been explored experimentally, it has been suggested we refer to descending control of pain as ‘descending control of nociception’ [10]. We will adopt this terminology in this review.

The descending control of nociception has an important adaptive function, allowing the adjustment of behaviour to different contexts to prioritize survival [4]. For example, if an animal is injured during a fight, the dampening of their nociceptive processing may increase the animal's fighting performance by ensuring they do not waste time or energy on responding to the injury. Likewise, when the animal has returned to safety, the descending controls can facilitate nociceptive processing, encouraging the animal to protect the injured location so that its healing is promoted. These arguments would make adaptive sense in any animal. Surprisingly, however, in the most speciose animal class, the insects, that are already known to have descending pathways for non-nociceptive behaviours (e.g. locomotion [11,12] and sexual behaviour [13,14]), such descending pain controls have been little investigated [15,16].

**Figure 1.**
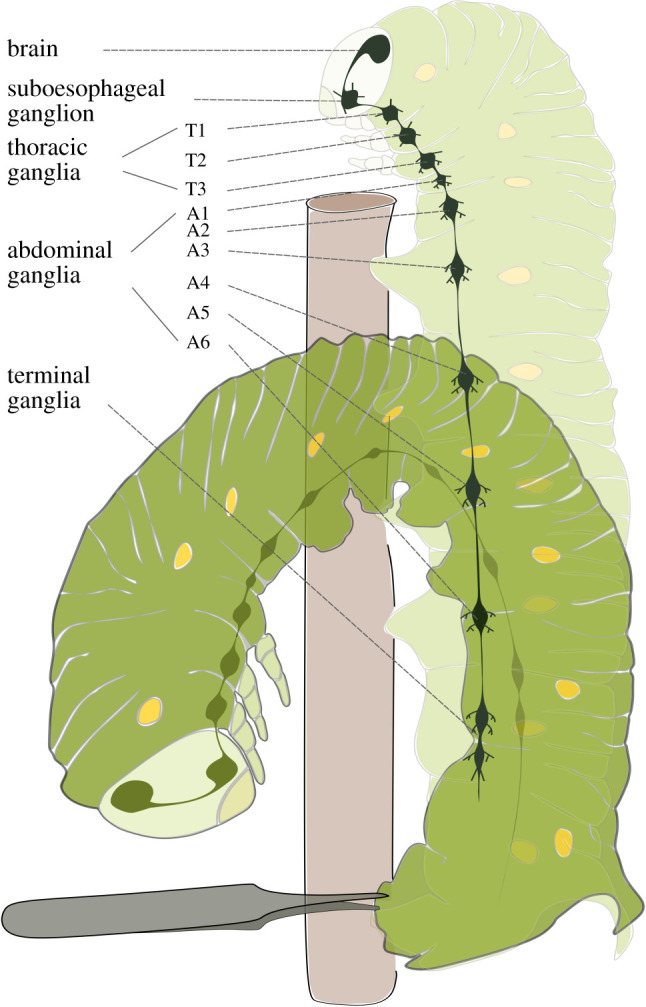
The defensive strike response and the nervous system of the tobacco hornworm (the larva of the tobacco hawkmoth *Manduca sexta*). This caterpillar shows a rapid bending response toward the site of the pinching stimulation on the terminal segment of the abdomen (source of the stimulation). The success of attackers such as birds that bite caterpillars can be reduced by the high velocity and precise targeting of the strike. This defensive strike response is faster and larger after repeated noxious stimulation and results in nociceptive sensitization [19]. Adapted from [19]. (Online version in colour.)

Nociception, and nociceptive behaviour, are well documented in insects [17,18]. Further, insect nociceptive processing can be modulated (e.g. [15,16]). For example, the tobacco hornworm (*Manduca sexta*) shows a defensive nociceptive behaviour in response to a noxious pinch, performing a rapid bending response toward the pinch site (figure 1), and this response can be sensitized by tissue damage [19,20]. However, the specific mechanisms and pathways of modulation of nociception in insects have only been partially uncovered, and it is not fully established whether the modulation involves the brain. In this review, we suggest that insects have descending modulation of nociception, based on behavioural and anatomical evidence.

## 2. Modulation of nociceptive responses by neural mechanisms outside the brain in insects

All insects exhibit nocifensive actions, a class of behaviours that occur in response to noxious stimuli and have the purpose of reducing exposure to the stimulus. An example of a nocifensive behaviour in fruit flies (*Drosophila melanogaster*) is the moving away from a floor heated to 46°C (e.g. [18]). Nociceptive sensitization occurs when the intensity of normal nocifensive behaviour is increased, or the threshold for the induction of the nocifensive behaviour is lowered [16]. In mammals, this sensitization can occur through molecular mechanisms at the site of damage [21] from activation of facilitatory projection neurons in the rostral ventral medulla [22], or in the dorsal horn in the spinal cord [23]. These mechanisms ultimately increase the nociceptive neurons' responsiveness to noxious stimuli.

Insects are capable of nociceptive sensitization, although the effect of descending control on this sensitization is unknown. For example, in larval *Manduca sexta*, the defensive strike response to a noxious stimulus is faster and greater after repeated noxious stimulation [19] (figure 1). Similarly, in fruit flies, injury of epidermal cells by ultraviolet light increases the speed of the flies’ withdrawal response from both sub-noxious and noxiously heated thermal stimuli [24,25]. Some of the molecular mechanisms underlying the sensitization of nociception in insects have been revealed. Peripheral mechanisms involve some of the same molecules responsible for human nociceptive sensitization. In fruit flies, like humans, signalling molecules including Hedgehog, tachykinin and tumour necrosis factor are involved in the sensitization of the nociceptors [21,25,26]. Also similarly to humans, central mechanisms have been suggested; e.g. in larval *Manduca sexta,* sensitization of the defensive strike response is associated with a reduction in firing threshold of the central connective nerve and can be blocked using N-methyl-D-aspartate receptor and hyperpolarization-activated, cyclic nucleotide-gated antagonists [20]. Further, in fruit flies, a loss of GABA inhibition in the ventral nerve cord causes nociceptive sensitization [24]. However, whether the brain is involved in sensitization in these studies remains unknown.

Inhibition, as opposed to sensitization, of nociceptive responses in insects has gained less attention. However, there is behavioural evidence of reductions in normal nocifensive behaviour in insects in certain situations. For instance, female mantids will consume the male during copulation, and the male appears to suppress his normal nocifensive behaviour to allow this [27]. This is probably because the male has fitness benefits from being consumed, as sacrificing his body as nutrition for the female increases the number, size and survivorship of the offspring [27]. Another example of inhibition of nocifensive behaviour in insects is how, in some cases, insects have been noted to act visibly ‘normal’ after injury, by continuing to feed or not altering their behaviour [28]. This evidence has been suggested to indicate the absence of pain in insects [29,30]; however, it is more likely that it demonstrates that insects can prioritize other behavioural needs and reduce the nocifensive behaviour in certain contexts [31].

**Figure 2.**
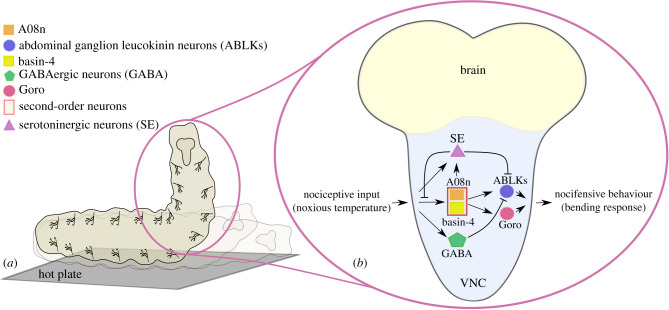
Nocifensive response to noxious stimulus and possible molecular pathways for inhibition of nociception in larval *Drosophila melanogaster*. (*a*) Nocifensive rolling: a corkscrew-like rolling response to noxious temperature that acts as a protective escape behaviour [17]. (*b*) Putative molecular pathway for inhibition of nocifensive behaviour in VNC (ventral nerve cord). Nociceptive input activates second-order neurons (SONs) such as A08n and Basin-4, serotonergic neurons and GABAergic neurons. SONs then activate abdominal ganglion leucokinin neurons and Goro neurons, which induce nocifensive behaviour. GABAergic and serotonergic neurons inhibit the activity of abdominal ganglion leucokinin neurons. Serotonergic neurons inhibit synaptic transmission between nociceptive input from nociceptors and SONs. (Online version in colour.)

The mechanisms behind inhibition of nociceptive responses are poorly understood in most insects. In fruit flies, there are second-order interneurons in the ventral nerve cord that are activated by nociceptors and integrate different sensory stimuli [32], as well as triggering nocifensive rolling behaviour in fruit fly larvae [33–35]. Two types of these interneurons, Basin-4 and A08n, are involved in the inhibition of nociceptive signalling in fruit fly larvae, via an inhibitory feedback loop with serotoninergic neurons (figure 2*b*) [34]. Basin-4 and A08n also activate neurons expressing the neuropeptide leucokinin in the abdominal ganglion of the fruit fly larva, which are required for escape behaviour from noxious stimuli [33], and these neurons express the serotonin receptor 5-HT1B [36]. This suggests that serotonin may also be able to inhibit nociceptive processing via leucokinin neuron signalling. Another mechanism of inhibition of nociception that has been uncovered is GABAergic neurons, which inhibit the activity of these abdominal ganglion leucokinin neurons [33]. These findings demonstrate that inhibition of nociception in insects is possible via molecular pathways (figure 2*b*). These peripheral and ventral nerve cord pathways may contribute highly to modulation of nociception in insects, but, on their own, they are unable to explain how stimuli that are processed in the brain (e.g. appetitive stimuli) are able to inhibit nociception, and how this inhibition could occur via descending controls from the brain.

## 3. Descending nociception controls in insects?

**Figure 3.**
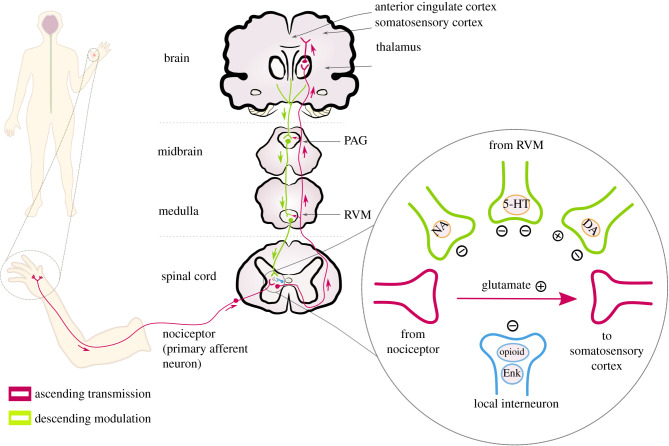
Human descending and ascending pain pathways. Primary afferent neurons project to secondary neurons in the dorsal horn of the spinal cord. Axons of the second-order neurons project to the thalamus, the rostral ventral medulla (RVM) and the periaqueductal grey (PAG). With their cell bodies in the thalamus, third-order neurons project to the somatosensory cortex to encode the sensory-discriminative aspects of pain. They also project to other areas, such as the anterior cingulate cortex, which are involved in the affective aspect of pain. These areas provide input to the PAG, which communicates with the RVM to send modulatory projections to the spinal cord. Such neurons influence enkephalin (Enk) interneurons which can inhibit the transmission of nociception through primary afferent neurons. Monoaminergic pathways, including serotonin (5-HT), dopamine (DA) and noradrenaline (NA), also have facilitatory and inhibitory modulatory effects. Adapted from [5]. (Online version in colour.)

Modulation of nociception in insects has been demonstrated behaviourally, and some of the molecular mechanisms underlying this have been identified (see above). In this section, we explore whether the modulation of nociception in insects can be activated by the brain, via descending controls. To explore this possibility, it is useful to contemplate how this pathway works in organisms better studied in this regard. In mammals, nociceptors transmit the information to the dorsal horn of the spinal cord, and the signal is then sent to the brain via ascending projection neurons [37] (figure 3). The periaqueductal gray in the midbrain receives nociceptive inputs, as well as inputs from cortical brain areas involved in pain processing, and transmits the signal to the rostral ventromedial medulla (RVM) [38]. The RVM projects to the dorsal horn and has distinct cell types which descend to the spinal cord and can inhibit or facilitate nociception [38].

Behavioural evidence suggesting the brain is involved in modulation of nociception exists in the American cockroach (*Periplaneta americana*), where the threshold required for nocifensive escape behaviour is increased after being stung in the suboesophageal ganglion in the brain by the parasitic jewel wasp (*Ampulex compressa*) [39–41]. This indicates that the insect brain can modulate nocifensive behaviour. Further support comes from the observation that nocifensive behaviour can be inhibited by stimuli processed in the brain, or by memories of such stimuli. For example, the taste of sugar processed in the fruit fly brain [42], and attraction to an odour that was previously associated with sugar suppresses normal avoidance of noxious stimuli in fruit flies [43]. Further, in bumblebees, the attraction to a sugar solution also suppresses normal avoidance of a noxious stimulus, but this can change depending on the context, specifically whether there is more concentrated sugar solution available elsewhere [44]. Since olfaction and gustation are processed in the insect brain, the effect of these on nociceptive behaviour should be mediated by descending neurons. Similarly, the processing of food-deprivation in the brain reduces the nocifensive jump response to noxious heat in fruit flies [45]; this apparently relies on the brain, as the same reduction is not observed in decapitated flies (although basic nociception is maintained) [45]. Taken together, behavioural evidence suggests that the insect brain can exert descending control over nociceptive processing.

Anatomical evidence also supports the existence of insect descending nociception controls. Studies have identified neurons that descend from the brain to the nerve cord and are involved in insect nociception. For example, in *Drosophila* larvae, axons descend from the brain to the Basin and Goro neurons, which mediate the nocifensive rolling response (a corkscrew-like roll in response to noxious stimuli [17,35]) (figure 2). In adult *Drosophila*, some neurons that connect the brain and ventral nerve cord express the protein ‘Straightjacket’, a calcium channel that mediates nociceptive hypersensitivity [24,46].

## 4. Characteristics of the putative insect descending nociception controls

**Figure 4.**
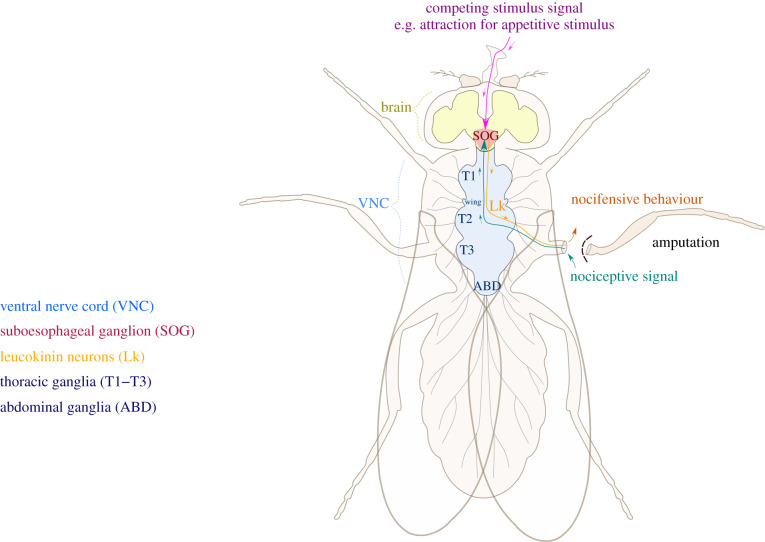
An example pathway for the putative insect descending nociception controls in the *Drosophila melanogaster* adult. Amputation of the middle leg causes nocifensive behaviour in fruit flies; this nocifensive behaviour can be reduced when the fly is hungry. This is mediated by leucokinin neurons (Lk). The nociceptive signal is transmitted to the ventral nerve cord (VNC), which can cause nocifensive behaviour, but is also transmitted along ascending fibres to the suboesophageal ganglion (SOG). A competing stimulus signal, such as attraction to an appetitive odour or food, is also transmitted to the suboesophageal ganglion. The appetitive and aversive signals are integrated here. Leucokinin neurons descending from the suboesophageal ganglion can suppress the nocifensive behaviour depending on the integrated information. (Online version in colour.)

Given the evidence discussed above, it is plausible that insects have descending nociception controls to modulate their nocifensive behaviour in certain contexts. Here, we discuss the putative chemical and anatomical characteristics of these controls. In mammals, many neurotransmitters and neuropeptides are involved in descending modulation of nociception, some having facilitatory roles, some inhibitory, and some both [47]. Opioid peptides are important for inhibitory descending modulation [38], so it has been suggested that insects' descending controls might also involve opioid signalling [48]. However, genomic studies have determined that insects do not have genes that code for opioid receptors or peptides [49–52]. Thus, it is more likely that another neuropeptide or neurotransmitter functions as the signalling molecule for the putative insect descending nociception controls. GABA is involved in mammalian descending modulation of nociception [53], and in fruit flies, it was found that a GABA agonist reduces nocifensive behaviour [54]. Another possibility is neuropeptide F, the insect homolog of the mammalian neuropeptide Y, that is involved in descending modulation of nociception [55]. Neuropeptide F suppresses nocifensive behaviour in fruit fly larvae through action on the nociceptor TRPA [56,57], however, this effect does not appear to generalize to honeybees [58]. Further, the peptide hormone cholecystokinin is involved in descending facilitation of nociception in mammals [59], and its insect orthologue Drosulfakinin is involved in stress-induced avoidance behaviours, but its effects on nociception and modulation of nociception in insects are unknown [60]. The neuropeptide somatostatin is also involved in modulation of nociception in mammals [61], and its insect orthologue, allatostatin-C, mediates modulation of nociception in insects [62]. Another candidate is leucokinin, a neuropeptide with many functions in insects, including the modulation of nocifensive behaviour (figure 4) [33,63]. There are leucokinin neurons that descend from the suboesophageal ganglia to the ventral nerve cord [33,64]. Importantly, these neurons are able to suppress nocifensive behaviour [33,45,63]. Specifically, they are required for the reduction of nocifensive behaviour in hungry fruit flies [45]. This is interesting because, as mentioned earlier, the jewel wasp modulates the American cockroach's nocifensive behaviour by stinging in the suboesophageal ganglia [39–41]. Since taste is also represented in the suboesophageal ganglia in insects [42,65,66], integration of competing stimuli may occur here. Leucokinin neurons may, therefore, project from the suboesophageal ganglion to modulate nociceptive behaviour according to certain contexts or stimuli. It is important to consider, however, that leucokinin is heavily involved in feeding regulation in insects [63], so this pathway may only explain modulation of nociception by hunger, and not other possible modulating stimuli such as stress. It is also worth noting that neurons in the fan-shaped body are likely involved in this pathway, as they are required for nociceptive avoidance [67], but how these neurons connect to other areas, neuron types and the nerve cord to elicit avoidance is unknown so far.

Other than the molecules discussed above, serotonin, noradrenaline (or octopamine in insects), neurotensin, tachykinins and glutamate are involved in mammalian descending modulation [68–72] but, in insects, either appear to have different or no effects on modulation of nociception [20,73–77]. There is probably more than one molecule involved in the putative insect descending controls of nociception, and some of them may be different from the molecules we have mentioned here.

## 5. The significance of insect descending nociception controls

The presence of descending nociception controls in insects is important and interesting for many areas of insect and human neuroscience. The descending control of nociception in humans can also affect pain perception, so it is conceivable that a form of pain perception exists in insects, and can be similarly modulated. This is certainly the accepted argument for mammals such as mice, where a reduction in nociceptive behaviour is accepted as equalling a reduction in pain (e.g. [78]). In insects, however, this argument is not widely acknowledged. This, perhaps, is because insect behaviour has often been viewed as governed largely by instinct, a view which is no longer tenable given what we now know about advanced cognition and emotion-like states in insects [79]. Nonetheless, insect behaviour toward injuries has been likened to robots [80] and various scholars have denied the existence of pain in insects [28,30,80].

The presence of descending controls makes it at least plausible that insects have painful experiences. Mammalian researchers quantify pain through measuring non-reflexive, complex and long-lasting changes to the animal's natural behaviour, which are likely mediated by descending controls [81]. For example, in rodents, reduced feeding [82], locomotion [82] and burrowing [83] behaviours are used as pain indicators. Thus, the examples of insects performing these kinds of behaviours, such as the ones discussed earlier, may support the idea of pain in insects. For example, insects show reduced attraction to appetitive stimuli if they have to also experience nociceptive stimuli [43,44]. Further, recent evidence demonstrating sentience-linked cognitive abilities in some insects supports this idea, as well as studies indicating pain perception in other invertebrates (e.g. [84–86]).

This is important morally, as insects are often subjected to potentially painful stimuli in research and farming [87]. The possibility of pain sensations in insects is also an important consideration for modelling human pain disorders. The fruit fly *Drosophila melanogaster* is currently used as a model organism for human pain research, because of similarities in the genetics and behavioural responses to human nociception [88]. The abnormal and persistent pain states in humans seem to occur due to dysfunction of descending pain controls [38], so, if insects have descending nociception controls, they could potentially be viable models for human pain disorders.

## 6. Conclusion

We have argued that insects have descending nociception controls similar to what is observed in vertebrates, based on behavioural, molecular and anatomical evidence. Behaviourally, changes to the insect brain can change their nocifensive behaviour, whether this change is physical manipulation (e.g. [36]) or the processing of motivational stimuli [43,45]. At a molecular level, insects have molecular pathways that can inhibit nocifensive behaviour, peripherally and centrally. Anatomically, insects have descending neuronal projections from the brain to the ventral nerve cord, where nocifensive behaviour is executed. Future research should aim to further characterise modulation of nocifensive behaviour, and whether this is associated with pain in insects, to clarify whether we should be affording ethical protection to insects in potentially harm-inducing settings, such as farming and research. Further, elucidation of the neuronal and molecular pathways of descending control of nociception in insects may lead to the use of insects as a model organism for human pain conditions involving dysfunction of descending control.

## Data Availability

This article has no additional data.
